# p27, The Cell Cycle and Alzheimer´s Disease

**DOI:** 10.3390/ijms23031211

**Published:** 2022-01-21

**Authors:** Ana García-Osta, Jinya Dong, María Jesús Moreno-Aliaga, Maria Javier Ramirez

**Affiliations:** 1 Neurosciences Program, Center for Applied Medical Research (CIMA), University of Navarra and IdiSNA, 31008 Pamplona, Spain; agosta@unav.es; 2 Department of Pharmacology and Toxicology, University of Navarra and IdiSNA, 31008 Pamplona, Spain; jdong@alumni.unav.es; 3 Center for Nutrition Research and Department of Nutrition, Food Science and Physiology, University of Navarra and IdiSNA, 31008 Pamplona, Spain; mjmoreno@unav.es; 4 CIBER Physiopathology of Obesity and Nutrition (CIBERobn), Carlos III Health Institute, 28029 Madrid, Spain

**Keywords:** cell cycle, Alzheimer’s disease, p27^Kip1^, neurogenesis, cytoplasm-nucleus shuttle

## Abstract

The cell cycle consists of successive events that lead to the generation of new cells. The cell cycle is regulated by different cyclins, cyclin-dependent kinases (CDKs) and their inhibitors, such as p27^Kip1^. At the nuclear level, p27^Kip1^ has the ability to control the evolution of different phases of the cell cycle and oppose cell cycle progression by binding to CDKs. In the cytoplasm, diverse functions have been described for p27^Kip1^, including microtubule remodeling, axonal transport and phagocytosis. In Alzheimer’s disease (AD), alterations to cycle events and a purported increase in neurogenesis have been described in the early disease process before significant pathological changes could be detected. However, most neurons cannot progress to complete their cell division and undergo apoptotic cell death. Increased levels of both the p27^Kip1^ levels and phosphorylation status have been described in AD. Increased levels of Aβ42, tau hyperphosphorylation or even altered insulin signals could lead to alterations in p27^Kip1^ post-transcriptional modifications, causing a disbalance between the levels and functions of p27^Kip1^ in the cytoplasm and nucleus, thus inducing an aberrant cell cycle re-entry and alteration of extra cell cycle functions. Further studies are needed to completely understand the role of p27^Kip1^ in AD and the therapeutic opportunities associated with the modulation of this target.

## 1. Cell Cycle

### 1.1. Cell Cycle and Cell Cycle Regulators

**Figure 1:**
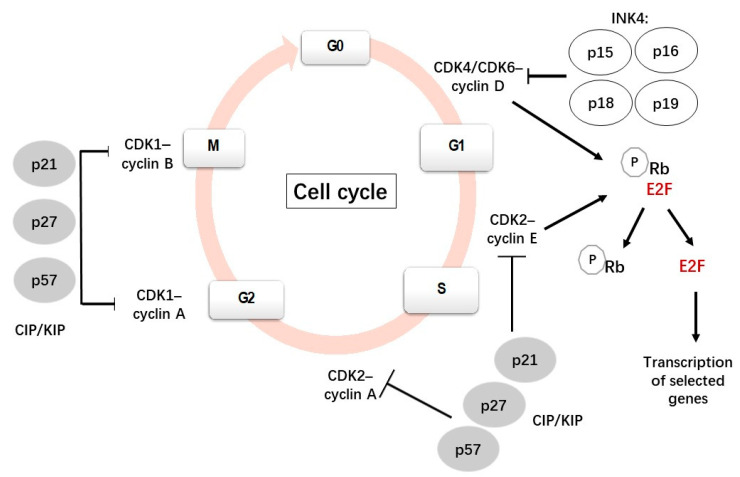
A scheme showing the canonical cell cycle and its main regulatory mechanisms [8,9].

The cell cycle is a complex and highly regulated process by which a cell divides into two cells. It is mainly constituted by four phases: Gap 1 (G1), DNA synthesis (S), Gap 2 (G2) and mitosis (M). The most important phases are S, when DNA replication occurs, and M, when the cell divides into two daughter cells. G1 is the first gap, preceding S, when cells prepare for DNA replication. During the second gap, G2 (after S), the cell prepares for mitosis (Figure 1). Many cells from the adult body exit from the cell cycle and stay in a quiescent state (G0). Several cells, such as neurons and most differentiated cells, do not enter again into the cell cycle but others, such as lymphocytes, exit and re-enter the cell cycle repeatedly throughout their lifetime [1,2,3].

The transitions between the stages in the cell cycle are finely coordinated by a family of serine/threonine kinases known as cyclin-dependent kinases (CDKs), which are active only when they are associated with their specific cyclins [4,5,6]. In contrast to cyclins, cyclin-dependent kinase inhibitors (CDKIs) inhibit the activities of CDKs. Phosphorylation and dephosphorylation events also regulate CDK activity. The ubiquitin-mediated proteolysis of cyclins and CDKIs has also been reported to be involved in cell cycle regulation [7].

Each CDK is able to associate with different cyclins with a specific role in each phase. For instance, when a cell is withdrawn from the quiescent state and enters the G1 phase, D-type cyclins (D1, D2, D3) bind and activate CDK4/6; these active complexes phosphorylate and inactive the retinoblastoma (Rb) protein family, which plays a negative regulation of the cell cycle. The phosphorylation of the Rb protein leads to the release of transcription factor E2F1 from its transcriptionally repressive complex, allowing the transcription of genes and the synthesis of the proteins necessary for DNA replication [10]. During the late G1 phase, cyclin E associates with CDK2 to progress through the S phase, subsequently binding to cyclin A during the early S phase and promoting DNA synthesis and replication [7,11].

Cyclin A also binds to CDK1; the CDK1/cyclin A complex is necessary for the transition from the G2 to the M phase. Cyclin A is degraded after prometaphase by a polyubiquitination-dependent mechanism [7]. CDK1 then associates with cyclin B to initiate the mitotic program by the phosphorylation of selected proteins. Subsequently, cyclin B is degraded by ubiquitin-mediated proteolysis, which is necessary to exit the M phase [7,12].

The activity of the CDKs/cyclin complexes can be inhibited by CDKIs, which are classified into two families: INK4 (p15^Ink4b^, p16^Ink4a^, p18^Ink4c^ and p19^Ink4d^) and CIP/KIP (p21^Cip1^, p27^Kip1^ and p57^Kip2^). Members of the INK family bind specifically to CDK4/6, preventing its interaction with cyclin D and regulating the quiescent state. By contrast, members of the CIP/KIP family have the ability to inhibit all cyclin/CDK complexes, including the CDK2/cyclin E, CDK2/cyclin A, CDK1/cyclin A and CDK1/cyclin B complexes [13,14]. p21 and p27 can bind and inhibit the cyclin E-CDK2 and cyclin A-CDK2 complexes and will stop the cell cycle at G1 [15]. In addition to the function of inhibiting cyclin-CDK activity, nuclear CIP/KIP proteins have alternative roles, such as regulators of nuclear import, apoptosis and cell motility, when they are located in the cytoplasm [16].

The deregulation of the cell cycle at any stage can result in a series of disorders, including cancer and neurodegenerative diseases among others [13]. In this context, it is relevant to mention cellular senescence, which is characterized by a stable cell cycle arrest, in which cells are unable to proliferate in response to mitogenic stimuli and optimal growth conditions [17]. Despite being in an arrested state, senescent cells are viable although they exhibit dramatic changes in gene expression and metabolic alterations as well as possessing a complex senescence-associated secretory phenotype [17]. In senescence cells, growth arrest occurs in the G1 and probably in the G2 cell cycle phases and seems to be mainly mediated by the p53/p21^WAF/CIP1^ and p16^INK4^/pRb pathways [18,19]. Growing evidence indicates that senescence cells contribute to aging and the development of age-related disorders, including chronic inflammation, Alzheimer’s disease and cardiovascular disorders [20,21,22].

### 1.2. Cell Cycle and Neurogenesis

Neurogenesis is the process of division of the neural stem cells (NSCs) and progenitor cells into daughter cells that migrate to corresponding brain areas and give rise to new neurons. It is linked to the cell cycle and is a crucial process in developing brains but it also takes place in adult brains where neurogenesis is required for the proper functioning of the mature neural network [23].

After the cell cycle withdrawal, newly generated neurons activate different programs of differentiation and migration [24]. Once the neurons are fully differentiated, they are considered post-mitotic cells and remain in a quiescent differentiated state (G0). They lose their capacity for cell cycle re-entry, which is probably promoted by changes in the expression of region-specific CDKIs [25,26]. However, the expression of cell cycle regulators persist after differentiation and are expressed in a manner that cannot be solely accounted for by differences in cell cycle rates [27], indicating that its role in neurogenesis is not only linked to cell cycle regulation. An increasing body of evidence has demonstrated that CDKIs have cell cycle-independent functions to coordinate cell cycle exit and neuronal differentiation, maturation and migration [28].

The majority of neuronal cells in the adult brain are fully differentiated and neurogenesis only persists in restricted brain areas; namely, the subventricular zone (SVZ) of the lateral ventricles and the subgranular zone (SGZ) of the hippocampal dentate gyrus (DG), the two main neurogenic niches [29].

The SVZ is the largest area in the adult brain where neurogenesis occurs; the resident neural stem cells (NSC) are primary progenitor cells that undergo numerous stages to finally originate mature neurons, astrocytes and oligodendrocytes [30]. The cells born in the adult SVZ (NSC, type B1 cells) express GFAP and can divide symmetrically and asymmetrically. Asymmetrical division commits to intermediate progenitors called type C cells, which after a mitotical division give rise to migratory neuroblasts (type A cells). The neuroblast, after one or two round divisions, exits the cycle, migrates towards the olfactory bulb and becomes newborn neuronal cells (post-mitotic immature neurons). A small group of these cells will survive and subsequently differentiate into granule cells [31]. A high expression of the CDK inhibitor p57 and p18-induced cell cycle arrest is required to maintain the production of undifferentiated NSC cells, inhibiting proliferation and differentiation [32,33].

The SVZ gives rise mostly to dopaminergic and γ-aminobutyric acidergic (GABAergic) interneurons that migrate from the rostral migratory stream and integrate into the olfactory bulb, playing an important role in the development of optimal olfactory circuitry where they participate in the processing of sensory inputs [34,35]. In physiological conditions, Type B1 cells also give rise to a few oligodendrocytes and astrocytes but in case of brain injury can produce more astrocytes that migrate to the damaged area [36].

In the SGZ, type 1 cells (radial glia-like precursors) divide asymmetrically, giving rise to transit amplifying cells (type 2 cells, GFAP-negative), which are highly proliferative. After several days of division, these generate type 3 cells that enter into a post-mitotic stage characterized by the expression of neuronal nuclei (NeuN) and calretinin (CR) [37,38]. Newly generated granule cells migrate radially into the granular cell layer where they develop dendrites and differentiate into glutamatergic granule cells, which are integrated into the neural circuitry of the hippocampus [39,40,41]. The degree of adult neurogenesis in the dentate gyrus correlates with the performance of animal models in hippocampus-dependent learning tasks [42,43], which suggests that neurogenesis is linked to memory and learning processes. Likewise, a decrease in neurogenesis in the DG during aging may contribute to age-dependent cognitive decline, tau hyperphosphorylation and a risk of Alzheimer’s disease [44,45,46,47].

Under physiological conditions, neurogenesis in the hippocampus is a dynamic and well-controlled process but it can be increased or decreased in different conditions, such as by physical activity or by irradiation, respectively [48,49,50]. Different pathological conditions, including psychiatric diseases, strokes or neurodegenerative disorders can also modulate neurogenesis [51,52].

In the last decades, new neurogenesis areas have been described in the adult mammalian brain in the hypothalamus, substantia nigra, striatum and amygdala [53,54,55,56]. However, these studies are still incomplete and more research is needed to confirm that neurogenesis takes place in these areas [57]. There are still many open questions related to adult neurogenesis. Discrepancies and differences between studies, probably due to technical problems, together with the very low number of newly generated neurons incorporated into the neuronal circuit make neurogenesis one of the most discussed topics in neuroscience.

To summarize, neurogenesis involves, in cell cycle terms, re-entering and exiting the cell cycle. The SVZ and DG are the two main neurogenic niches. The modulation of cell cycle components is not only related to the number of cells (proliferation) but also to cell linage differentiation.

### 1.3. Cell Cycle and AD

Alzheimer’s disease (AD) is a neurodegenerative disease and the most common cause of dementia in late life. Most of the people affected are over 65 years of age and the prevalence raises dramatically between 65 and 85 years [58]. Brain lesions start long before the onset of cognitive problems; this is named the preclinical phase of the disease. The disease progresses with the deposition of β-amyloid (Aβ) in senile plaques and the formation of intracellular neurofibrillary tangles (NFTs) composed of the hyperphosphorylated tau protein. The hippocampus is one of the brain areas that earliest exhibits the damage and as the disease progresses, it spreads to other cortical areas (frontal and entorhinal cortex and another limbic areas), triggering a significant reduction in neuronal density in various regions [59,60]. When a massive loss of neurons takes place, the disease becomes symptomatic, starting with a slight cognitive decline of recently acquired information. It becomes worse with time, severely affecting cognitive dysfunction and other abnormal behaviors. In the late stage of the disease, the brain function continues to decline and the disease affects the autonomy of these patients [61]. The diagnosis of AD is based on different tests but a complete certain diagnosis can only be achieved by a post-mortem observation of the brain parenchyma and the presence of senile plaques and neurofibrillary tangles made up by the hyperphosphorylated tau protein (p-tau) [62].

Despite progress in the field, the precise pathogenic mechanisms of the neurodegenerative processes leading to AD remain elusive. In 1990, patients with Down syndrome showed AD-like dementia symptoms, starting the hypothesis that the aneuploidy (chromosome 21 trisomy) was causal to AD and a mutation in the genes involved in the cell cycle could lead to AD development [63]. More recent evidence, including -omics data from AD patients, has shown that an abnormal activation of the cell cycle components can contribute to neuronal death in AD [64,65,66,67,68]. Furthermore, these cycle event alterations are found in the early disease process of AD before significant pathological changes occur [69,70,71,72]. These data suggest that a group of neurons newly enter a defective cell cycle and most of them cannot progress to complete their cell division, thus undergoing apoptotic cell death [73]. Despite other studies reporting conflicting results [74,75], solid evidence supports the idea of increased aneuploidy in the neurons of AD patients. Interestingly, 13 out of the 37 genes that are frequently mutated in AD are functionally involved in the regulation of the cell cycle or mitosis (review in [76]).

p53 is a transcription factor that regulates the expression of the genes involved in critical cellular functions, such as cell cycle re-entry, DNA damage repair, developmental differentiation and senescence [77]. It has the ability to promote cell cycle arrest in several cell types and apoptosis in others. It is known as the “guardian of the genome” to limit the deleterious consequences of mutation [78]. During the development of the central nervous system (CNS), p53 is expressed in neuroblasts and is downregulated when migrating neurons reach their place [79]. In physiological conditions, p53 is maintained at low basal levels and its dysfunction has detrimental consequences, including cancer, metabolic disorders and neurodegenerative diseases. Elevated p53 immunoreactivity has been observed in sporadic and familial AD and in animal models [80,81]. p53 expression increases in parallel with an intracellular accumulation of Aβ [82] and induces tau phosphorylation in HEK293a cells [81]. It has been demonstrated that p53 oligomerizes in AD brains and interacts with tau oligomers, triggering the loss of the nuclear p53 function and contributing to AD pathology [83].

Interestingly, regulators of the cell cycle—including CDKs, CDKIs and p53—also regulate the autophagic processes [84,85], indicating that autophagy is intimately connected to the cell cycle [86]. Autophagy is also a basic physiologic process crucial to maintaining cellular homeostasis by clearing protein aggregates. AD, in common with other neurodegenerative diseases, is linked to the accumulation of misfolded proteins, suggesting that autophagy dysfunction probably contributes to AD pathogenesis [87,88,89].

Various studies have reported the overexpression of cell cycle protein regulators, such as CDKs, in the vicinity of NFTs and senile plaques in the post-mortem brains of AD patients [68,90], indicating that the overexpression of the regulators of the cell cycle may be a characteristic feature of AD. Consistently, the administration of CDKIs (inhibitors of CDK1, 2, 4, 5, 7, 9) ameliorated AD symptoms in animal models [91,92,93,94,95,96], corroborating that the cell cycle and mitosis play an important role in AD development and could become important targets of drug research. In this sense, CDKIs have been suggested to be of potential relevance in the treatment of AD [93].

Altogether, the connection between neuronal loss and cell cycle re-entry in AD has been described. Although the underlying mechanism of cycle re-entry in AD is not completely understood, the role of Aβ as a trigger of this aberrant cell cycle re-entry in post-mitotic neurons can be hypothesized, which in turn can initiate apoptotic neuronal death.

## 2. p27^Kip1^

### 2.1. p27^Kip1^ and Its Role in the Cell Cycle Progression

The cyclin-dependent kinase inhibitor p27^Kip1^ (named p27) participates in many biological processes, such as cell proliferation, differentiation, migration and apoptosis [97]. p27 has been extensively studied for its role in cancer as a tumor suppressor [98]. It plays fundamental roles in all phases of the cell division cycle. Specifically, nuclear p27 inhibits cell cycle progression and makes the cell susceptible to quiescence, apoptosis and/or senescence. Extensive studies have been conducted in this regard. p27 is expressed in the quiescent stem cells of the adult hippocampus and is induced in immature neurons upon differentiation, suggesting that p27 can act as a controller of the cell cycle exit and differentiation, keeping adult hippocampal stem cells out of the cycle [99]. Hörster et al. [100] analyzed the role of p27 in the transition of precursor cells to post-mitotic maturation in adult hippocampal neurogenesis. In the DG, p27 shows a strong nuclear expression in early post-mitotic neurons (type 3 and a few type 2) and it has a lower or an absent expression in radial glia-like precursor cells (type 1) and type 2 cells with no expression in granule cells. Thus, the p27 nuclear function may have a transitory role in late proliferative and early post-mitotic phases of neurogenesis. Neurogenesis was abrogated in p27^−/−^ mice whereas the content of proliferating cells was promoted [100]. Concerning neurogenesis, p27 plays an important role in adult brain neurogenesis and the deletion of p27 impaired neurogenesis due to the increase of intermediate progenitor cells [101,102].

Mechanistically, p27 prevents the G1/S transition by repressing the CDK2-cyclin E and CDK4-cyclin D complexes [103]. It also participates in the control of the G2 and M phases [103,104]. A depletion of p27 protein in the late G1 phase promotes the transition to the S phase [105], allowing cell division progress. p27 protein levels are controlled through the regulation of translation and ubiquitination-dependent protein degradation. The levels of p27 decrease when the cells exit the quiescent state due to this ubiquitin-mediated degradation but the levels of p27 can also increase in response to anti-proliferative signals, such as serum starvation [106].

In summary, p27 is a key regulator of cell proliferation. When p27 binds to and inhibits cyclin-CDK, it arrests the cell cycle and opposes cell cycle progression.

### 2.2. Extra Cell Cycle Regulatory Functions of p27

To control the progression of the cell cycle in the nucleus, several cell cycle-related proteins, including p27, have an extra cell cycle regulatory function after exiting from the cell cycle [107]. These functions include regulating cytoskeletal organization, proliferation, differentiation and apoptosis. In most tumors, nuclear p27 regulates the cell cycle, which leads to the growth inhibition of cancer cells whereas in the cytoplasm, it is oncogenic. In cortical neurons treated with Aβ42, the overexpression of p27 induces apoptosis through the stabilization of the CDK5-cyclin D complex as this stabilization prevents the interaction of CDK5-p35 [108].

The activity of p27 is mainly dependent on post-translational modifications, such as phosphorylation and others (for example, ubiquitination and acetylation), on different residues and on their subcellular (nuclear vs. cytoplasmic) localization [104]. The phosphorylation of p27 determines its degradation by the ubiquitin system and the sequestration in the cytoplasm, precluding its nuclear function as a CDK inhibitor [109]. p27 is phosphorylated on many sites, including tyrosine (Tyr(Y)74,88,89), threonine (Thr157,187,198) and Ser10 (Table 1).

**Figure 2:**
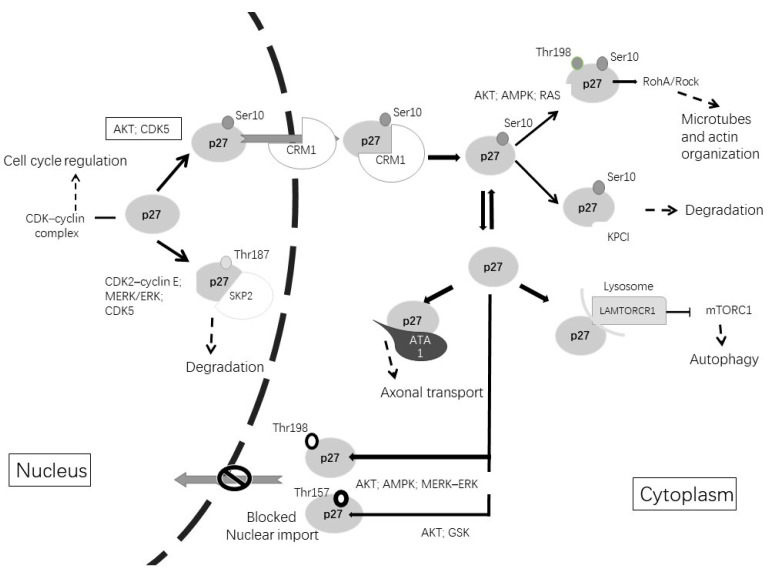
Post-translational modifications of p27^Kip1^ lead to changes in its function and alter its subcellular localization. The phosphorylation of p27^Kip1^ at Thr187 promotes its degradation in the nucleus. Phosphorylation of p27^kip1^ at Ser10 promotes exportation to the cytoplasm by CRM1. Once in the cytoplasm, p27^Kip1^ can be degraded or can undergo different phosphorylations. Phosphorylation at Thr157 or Thr198 avoids nuclear reimportation. Cytoplasmic functions include microtubules and actin organization, axonal transport or involvement in autophagy.

As shown in Figure 2, when the cell enters the G1 phase, the phosphorylation of p27 on Ser10 by AKT (protein kinase B), CDK5 or KIS (kinase interacting stathmin) promotes the exportation of p27 from the nucleus to the cytoplasm by CRM1 (chromosome region maintenance 1), a carrier protein for nuclear export [110]. When p27 stays in the cytoplasm, Ser10 phosphorylation allows KPC (ubiquitylation promoting complex)-dependent degradation. Furthermore, CDK5 stabilizes p27 through Ser10 phosphorylation, increasing the p27 protein in the cytoplasm. Therefore, CDK5 is an unconventional cyclin-dependent kinase and is neuron-specific, able to phosphorylate p27 both at Ser10 in the N-terminal as well as at Thr187 in the C-terminal [5,111]. Other phosphorylation sites are at Thr157 and Thr198, which avoid nuclear import and result in the cytoplasmatic location of p27. This phosphorylation can be induced by AKT, AMPK, p90 ribosomal protein S6 kinases, serum glucocorticoid-inducible kinase (SGK) or PIM (proto-oncogene serine/threonine-protein kinase) [111,112,113].

In the cytoplasm, p27 associates with microtubules and it is required for proper axonal transport, neuronal migration and dendritic spine formation [114,115,116]. The phosphorylation of p27 at Thr198 by RSK1 induces the interaction with RhoA and a subsequent reduction in RhoA-GTP binding, promoting cell migration and motility [118]. In cortical interneurons, p27 regulates nucleokinesis and promotes tangential migration and neurite branching by controlling the activity of myosin II through the Rho kinase pathway [119]. Studies in Drosophila and in rodents revealed that p27 regulates the axonal transport of vesicles and organelles through the stabilization of alpha-tubulin acetyltransferase 1 (ATAT1), which promotes the acetylation of microtubules. The neuronal depletion of p27 results in axonal transport defects that can be rescued by treatment with histone deacetylase inhibitors that increase the levels of tubulin acetylation [116].

All these alterations in the cytoskeleton and impaired axonal transport play an integral role in the pathogenesis of AD. On the one hand, the cytoskeleton is crucial for dendritic spine formation, which is necessary for a correct synaptic activity. On the other hand, axonal transport is a process that uses microtubules to deliver diverse cargoes (including organelles, mitochondria and vesicles) along the axon, which is essential for nerve development, function and survival [120,121,122].

To summarize, p27 has several extra cell cycle regulatory functions in addition to controlling the cell cycle. These functions include regulating cytoskeletal organization, proliferation, differentiation and apoptosis. The subcellular localization and activity are dependent on post-translational modifications.

### 2.3. p27 and AD

A few studies have analyzed a purported relationship between the alterations in p27 and AD. In an immunohistochemical study, it was found that the levels of cytoplasmic p27 and phosphorylated p27 (Thr187) were increased in the hippocampal pyramidal neurons in AD compared with age-matched control subjects [123]. Interestingly, the increase in phosphorylated p27 (Thr187) overlapped with the tau-positive neurofibrillary pathology, including neurofibrillary tangles, dystrophic neurites and neuropil threads, indicating a possible direct implication of phosphorylated p27 in AD pathogenesis [123]. However, an increase in p27 was observed in both tangle-bearing as well as tangle-free neurons, suggesting that an increase in p27 was not directly related to the pathology in AD. It was suggested that phosphorylation at Thr187 could be associated with the activation of either the cyclin E/CDK2 or MAPK/ERK (mitogen-activated protein kinase) pathways.

In another study, it was reported that the half-life of the p27 protein in lymphoblasts from AD patients was markedly reduced compared with those in healthy patients [124]. In this study, an increased phosphorylation of p27 at Thr187 mediated by the Ca^2+^/CaM-dependent overactivation of PI3K/AKT was suggested to be responsible for an enhanced degradation of p27 in AD cells. The degradation of p27 favors the progression of the cell cycle and an enhanced cell proliferation [124]. It is of note that these cell cycle disturbances might be considered to be systemic manifestations that mirror the pathological changes that take place in the brain. For instance, the overactivation of the pro-survival PI3K/AKT signaling pathway in AD brain that takes place to protect neurons [125] can lower the levels of p27 and, in post-mitotic neurons, can eventually lead to neuronal death [126].

Regarding animal models, increased levels of the p27 protein were found in the cerebral cortex of APPswe/PSΔE9 mice compared with age-matched controls [127]. In this case, the levels of p27 were regulated through a post-transcriptional mechanism mediated by the downregulation of the microRNA 222 (miR-222). This particular non-coding small RNA bound to the 3′-UTR of p27, triggering its translational repression [127].

The role of Aβ42 in the regulation of p27 levels and/or phosphorylation in AD that may lead to apoptosis has been speculated. In cortical post-mitotic mature neurons, p27 promoted neuronal apoptosis in the presence of Aβ42 [108]. These authors suggested that Aβ42 stabilized the CDK5-cyclin D1 complex with a subsequent decrease in CDK5, preventing its interaction with its neuron-specific activator p35. A depletion of p27 restored the CDK5-p35 interaction, inhibited the MAPK/ERK pathway and cyclin D1 expression and avoided the neuronal apoptosis mechanism. Aβ42 and other neurotoxic agents promoted p35 proteolytic cleavage to p25. It has been shown that p25 accumulates in the brain of AD patients and induces the mislocalization and aberrant activation of CDK5 that can promote tau hyperphosphorylation, leading to neuronal cell death [68]. Interestingly, the inhibition of CDK5 is able to reduce cell death in Aβ42-treated neurons.

A relation between autophagy and p27^Kip^ has also been reported [117,128,129]. Autophagy is a catabolic process directed to degrade and recycle damaged organelles and misfolded proteins. The most predominant form is macroautophagy, in which a double membrane structure (named autophagosomes) surrounds the damaged organelle or protein and is exposed to lysosomes for degradation [130]. The dysfunction of autophagy is suggested to lead to the accretion of noxious proteins, such as Aβ42 or phosphorylated tau in the AD brain [131]. It has been suggested that p27 can drive apoptosis or autophagy following AMPK activation. AMPK phosphorylation of p27 on Thr198 promotes p27 protein stability and increases the levels of cytoplasmatic p27, resulting in greater autophagy and fewer apoptotic processes [98]. Other authors have proposed that the mechanism responsible for p27-controlled autophagy is regulated via mTOR (mammalian target of rapamycin) [117]. As reviewed [131], mTOR activation is an important fact in AD, which can lead to the impairment of autophagy. A fraction of p27 is recruited into the lysosomes where it binds to the LAMTOR1 subunit, avoiding the regulator-rag complex assembly, thus preventing mTORC1 activation [117].

**Figure 3:**
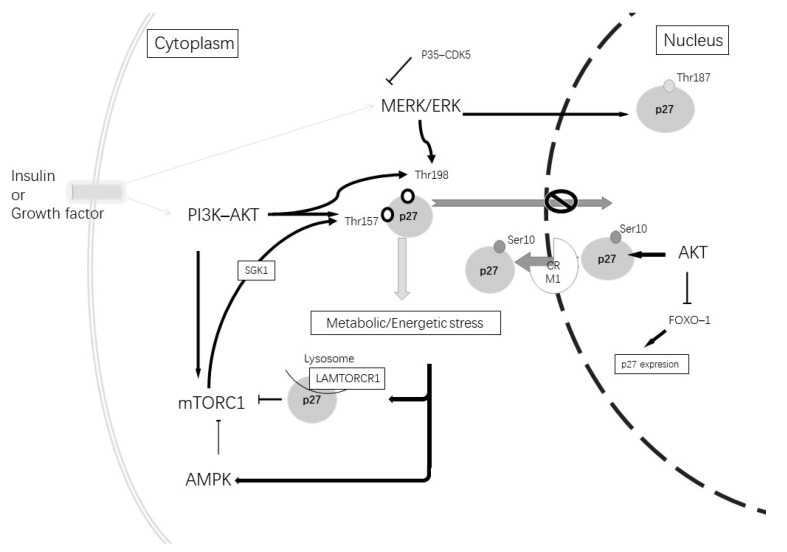
Insulin or growth factors can stimulate tyrosine kinase inducing the activation of the MERK-ERK and PI3K-AKT pathways. The activation of the MERK-ERK pathway stimulates p27^Kip1^ phosphorylation at Thr187, which induces its degradation. Its phosphorylation at Thr198 produces p27^Kip1^ cytoplasmic retention. The MERK-ERK pathway can be inhibited by p35 and the CDK5 complex. The activation of the PI3K-AKT pathway causes mTORC1 activation and causes phosphorylation at Thr157 through SGK1. Furthermore, AKT can phosphorylate at Thr198 and Thr157, which causes p27^Kip1^ cytoplasmic retention. AKT at a nuclear level phosphorylates Ser10, promoting p27^kip1^ nuclear export and can induce the inhibition of FoxO1 TFs and repress the gene expression of p27^Kip1^. A metabolic or energetic stress condition induces mTORCR1 inhibition by the induction of p27^Kip1^ binding to LAMTORCR1 and AMPK pathway activation. In addition, AMPK phosphorylates p27^Kip1^ at Thr198.

In relationship with both AD alterations and autophagy, another important cellular pathway to consider is PI3K/ATK. Alterations in this pathway have been described in AD in correlation with diabetes mellitus type 2 [132,133] and can affect p27 expression and phosphorylation (Figure 3). Altered insulin signaling increases phosphorylated PI3K and the activation of AKT, which leads to the phosphorylation of mTORC1, inhibiting autophagy. Therefore, AKT regulates the p27 nuclear export and sequestration of p27 in the cytoplasm [109]. Furthermore, mTORC1 activates SGK1, a protein kinase component of the PI3K-AKT pathway, which phosphorylates p27 at Thr157; therefore, it is retained in the cytoplasm [134].

In summary, the re-expression of cell cycle-related proteins in AD has been shown. Stimuli, such as Aβ42, might lead to an abortive re-entry into the cell cycle and, ultimately, neuronal degeneration. The dysregulation of the cell cycle, therefore, might play a crucial role in the pathogenesis of AD, which may provide a novel mechanistic basis for therapeutic interventions.

## 3. Concluding Remarks

In conclusion, cell cycle activation plays a role in the pathophysiology of neurodegenerative diseases, including AD, and cell cycle re-entry is considered to be one of the key causes of neuronal death in the illness. Therefore, the cell cycle provides a potential target for treatments and therapies.

The cell cycle coordination needs a complex interplay between the levels of various cyclins and CDKs at different checkpoints. The involvement of aberrant cell cycle re-entry in the pathogenesis and progression of neurodegenerative diseases could be inhibited by the action of cyclin and CDK-specific inhibitors. However, CDKI inhibitors, such as p27 not only play a key role in cell cycle control but also post-transcriptional modifications, such as phosphorylation, contribute to extra cell cycle functions or even increase the degradation. In the nucleus, p27^Kip1^ inhibits the cell cycle progression and makes the cell susceptible to quiescence and apoptosis. However, in the cytoplasm, p27^Kip1^ has been found to promote cellular resilience through autophagy and anti-apoptotic mechanisms [98]. Altogether, all these nuclear and extra-nuclear functions allow a correct neurogenesis, maintaining a quiescent state in the post-mitotic neurons. As already detected in AD, the attempt of neurons to re-enter the mitosis in response to external stimuli (such as Aβ42, p-tau or insulin resistance) leads to an abortive re-entry into the cell cycle and, ultimately, neuronal degeneration. In this sense, the increase in both p27 and phosphorylated p27 (Thr187) in the AD neurons may reflect an effort of the susceptible neurons to stop the cell cycle. Further studies will shed light on the role of p27 at the cytoplasmic level to better understand the involvement of p27 in the mechanisms related to AD progression.

One of the pending affairs in AD is to find early biomarkers for sporadic AD, which could identify patients before substantial pathology develops. Nowadays, by the time the disease is diagnosed, the pathology has spread, causing irreversible damage and functional disability. The fact that it is possible to detect changes in the p27 protein levels in lymphocytes from AD patients, which could mirror changes thought to occur in the brain prior to neurodegeneration, could offer new venues for diagnostic tools. Furthermore, the identification of therapeutic targets directed to normalize the cellular content of p27, thus reducing the activity of CDKs and cell cycle activation, could also open new venues for AD treatment. In this sense, CDKIs, by preventing CDK inactivation, might constitute a promising venture for the treatment of AD. However, although several CDKIs have been reported in the literature to treat other diseases, such as cancer, few studies have been conducted to investigate their effects against neurodegeneration. Not only that but also various side effects may arise due to the non-specificity of CDKIs, which may affect several CDKs, leading to controversial opinions towards its use [93]. Therefore, achieving a higher selectivity might be considered to be one of the most important aspects in the field of CDKI development.

## Figures and Tables

**Table 1:** Phosphorylation sites of p27^Kip1^: its role in cellular localization and function.

Subcellular Localization	Phosphorylate Site	Phosphorylated by	Function
Nuclear	Thr187	CDK2–cyclin E; MERK/EK; CDK5	Degradation by Skp2 [109]
Nuclear, cytoplasmatic	Ser10	AKT; CDK5	Nuclear export [110,111]
Cytoplasmatic	Thr198 or/and Thr157	AKT; AMPK; GSK; MERK–ERK	Blocked nuclear import [109]
Cytoplasmatic	Thr198 and Ser10	AKT; AMPK; CDK5; MERK–ERK	Microtubes and actin organization [114,115]
Cytoplasmatic	Independent of phosphorylation	–	Axonal transport via stabilization ATA1 [116]
Cytoplasmatic	Independent of phosphorylation	–	Increment autophagy via LAMTOR1 [117]
